# Augmented surgical decision-making for glioblastoma: integrating AI tools into education and practice

**DOI:** 10.3389/fneur.2024.1387958

**Published:** 2024-06-07

**Authors:** Melike Mut, Miaomiao Zhang, Ishita Gupta, P. Thomas Fletcher, Faraz Farzad, Divine Nwafor

**Affiliations:** ^1^Department of Neurosurgery, University of Virginia, Charlottesville, VA, United States; ^2^Department of Electrical and Computer Engineering, Department of Computer Science, University of Virginia, Charlottesville, VA, United States

**Keywords:** glioblastoma, artificial intelligence, machine learning, deep learning, surgical decision making, connectomics, resection

## Abstract

Surgical decision-making for glioblastoma poses significant challenges due to its complexity and variability. This study investigates the potential of artificial intelligence (AI) tools in improving “decision-making processes” for glioblastoma surgery. A systematic review of literature identified 10 relevant studies, primarily focused on predicting resectability and surgery-related neurological outcomes. AI tools, especially rooted in radiomics and connectomics, exhibited promise in predicting resection extent through precise tumor segmentation and tumor-network relationships. However, they demonstrated limited effectiveness in predicting postoperative neurological due to dynamic and less quantifiable nature of patient-related factors. Recognizing these challenges, including limited datasets and the interpretability requirement in medical applications, underscores the need for standardization, algorithm optimization, and addressing variability in model performance and then further validation in clinical settings. While AI holds potential, it currently does not possess the capacity to emulate the nuanced decision-making process utilized by experienced neurosurgeons in the comprehensive approach to glioblastoma surgery.

## Introduction

High-grade gliomas (HGG) stand as the most prevalent and deadly primary malignant brain tumors in adults. Among these, glioblastoma (GBM) is the most frequent malignant brain tumor, constituting 14.2% of all tumors and 50.9% of all malignant tumor. In the United States, its incidence is reported at 3.27 per 100,000 population. Typically affecting individuals with a median age of 65 years, GBM exhibits a remarkably poor overall survival, despite the implementation of combined radio-chemotherapy. Survival durations typically range between 15 and 17 months, with a median survival of only 8 months (1).

The decision-making process for surgical interventions in patients with GBM is inherently challenging, suffering from a lack of clear guidelines, particularly regarding the choice between biopsy and resection. Surgeons are confronted with the intricate task of assessing resectability, carefully balancing the advantages of decompression and cytoreduction against the potential neurological consequences. Navigating the diverse clinical landscape characterized by varied glioma molecular subtypes, tumor locations, sizes, eloquent area involvement, and co-existing medical complexities poses a formidable challenge (2).

Moreover, while individual surgeons manifest considerable variability in their clinical judgment regarding surgical resectability, aggregated responses from a large number of surgeons prove to be more consistent and predictive. Sonabend et al. (3) demonstrated a robust correlation between surgical resectability in GBM patients and defined GBM resectability through the wisdom of the crowd. Their study, derived from the pooled responses of 13 surgeons and the percentage of contrast-enhancing tumors, revealed a significant correlation. Despite notable variability in individual surgical goals among neurosurgeons, the resectability index, derived from the pooled responses of surgeons, exhibited a strong correlation with the percentage of contrast-enhancing residual tumor.

Recognizing cognitive biases and understanding decision-making processes are pivotal in enhancing patient care, especially in neurosurgery where errors carry significant consequences. The Dual Process Theory (DPT) illuminates two cognitive processes – analytical and rapid, unconscious and biased implicit processes. Despite the prevalent belief in analytical decision-making, much of daily clinical decisions are influenced by the rapid and unconscious implicit system, leading to inherent human biases (4). The growing adoption of AI, machine learning (ML), and deep learning (DL), particularly with the analysis of extensive datasets, presents a compelling foundation for developing an AI-based prediction and probably decision-making systems.

AI encompasses the use of computers and technology to mimic intelligent behavior and critical thinking, similar to humans. Within AI, ML is a subset that employs methods capable of automatically identifying patterns in data for predicting future data or making decisions under uncertainty. The learning process in ML can take the form of supervised or unsupervised learning. Supervised learning establishes a pattern connecting inputs to outputs using a labeled set of input–output pairs, for tasks like classification and regression. In contrast, unsupervised learning extracts patterns or structures from input data without relying on labeled data or predefined outcomes. Such learning algorithms extract patterns from input data without predefined outcomes, revealing insights that may not be immediately apparent, for example, personalized treatment strategies and hidden disease patterns. ML/AI techniques have become increasingly important in healthcare applications, providing innovative solutions to various challenges in clinical settings (Table 1). For example, supervised learning methods, such as classification and regression, are often developed for disease diagnostics and prediction, as well as stratifying individuals based on risk factors. Notably, ML algorithms have been shown to analyze medical imaging data using Convolutional Neural Networks (CNNs)-based networks, enabling accurate and efficient detection of anomalies in radiology or pathology images (11, 12). The integration of DL, ensemble methods, and reinforcement learning further improves the capabilities of ML applications in healthcare, paving the way for more precise diagnostics, optimized treatment strategies, and improved patient outcomes. As these techniques continue to evolve, they hold the potential to transform healthcare delivery, making it more personalized, efficient, and data driven.

**Table 1:** Examples of AI tools for glioma imaging and prediction.

Focus of the Study	Methodology	Evaluation Technique	Outcome/Performance	Remarks
Predicting surgical resectability (5)	Artificial NN	Receiver Operator Characteristic (ROC) curves; Area Under Curve (AUC) and accuracy calculations	AUC of 0.87–0.92; Accuracy of 83–87%	Compared against logistic regression and a standard grading system
Differentiation between non-enhancing tumor and vasogenic edema (6)	Support Vector Machine (SVM) Classifier	ROC analysis	Misclassification error reduced to 2.4% with post-processing	Utilized T1 perfusion MRI parameters
Prediction of tumor recurrence or necrosis (7)	Convolutional NN combined with Long Short-Term Memory (CNN-LSTM)	AUC, AUPRC, F1-score	AUC of 0.83; AUPRC of 0.87; F1-score of 0.74	Combined MRI data and clinical features
Differentiating vasogenic edema from non-enhancing tumor (8)	DL with multimodal MRI	Accuracy, sensitivity, specificity	Accuracy up to 90.3%; sensitivity and specificity significantly better than neurosurgeons	Histology examination of the resected tissue for validation
Differentiation between pseudo-progression and true progression (9)	DL model	ROC analysis; Leave-one-out cross-validation	AUC up to 0.92; Accuracy up to 87% for predicting PsP	Utilized preoperative and intraoperative MRI data
Predicting regions of local recurrence in GBM (10)	ML with voxel-based radiomic features	AUC, Accuracy, Precision, Recall, F1 Score, Cohen’s Kappa	AUC of 0.81 ± 0.09, Accuracy of 0.84 ± 0.06, Precision of 0.48 ± 0.24, Recall of 0.76 ± 0.22, F1 Score of 0.53 ± 0.17, Cohen’s Kappa of 0.45 ± 0.18	Utilized postoperative MRI data

NN, Neural network; DL, Deep learning; ML, Machine Learning; MRI, Magnetic resonance imaging.

In recent years, AI applications in medicine, spanning various medical specialties, have experienced significant growth. A notable advancement in radiology involves the transformation of biomedical images, such as magnetic resonance imaging (MRI), into mineable data, coupled with their analysis using AI techniques—commonly referred to as “radiomics” (13). Radiomics aims to extract quantitative and reproducible information from diagnostic images, focusing on the analysis of complex patterns that may be challenging for the human eye to discern or quantify. This approach entails capturing properties of tissues and lesions, including shape and heterogeneity. In the realm of brain tumors, radiomic research is dedicated to identifying features that describe the tumor and its microenvironment. The overarching objective is to construct predictive models for various tumor variables and patient outcomes. Notably, these radiomic models surpass their clinical counterparts in performance, offering predictions for outcomes in GBM, such as overall survival (OS), progression-free survival, molecular subtypes, and genetic alterations (13). The literature is increasingly featuring AI tools designed to predict the resectability of GBM. Table 1 summarizes some of the methodologies, evaluation techniques, and outcomes for AI/ML models in glioma imaging and prediction.

The main goal of this study is to conduct a thorough literature review, concentrating on current AI tools. The objective is to analyze a wide range of predictive factors, both tumoral and non-tumoral, along with their potential interactions. This review explores the use of AI tools in the context of surgical decision-making for GBM patients, with a specific focus on predicting resectability and surgery related early postoperative neurological outcomes.

## Methods

The literature search for the study involved the use of three bibliographic databases (Pubmed, Web of Science, Google Scholar, and Scopus) from their respective inception date to January 2024. The search term constructs used in all three databases were “connectomics” or “radiomics” and “AI” or “deep learning” or “machine learning” and “glioblastoma” and “surgical” or “decision making.” This search string generated a total of 117 articles. Two investigators (AMM and FF) independently screened the titles, abstracts, and full texts retrieved from all three databases to determine the eligibility of the studies. Publications outside the scope of neurosurgery, preclinical studies, non-peer reviewed, duplicates, and GBM/HGG studies focused on AI or ML at the molecular level were excluded from the study. The study’s inclusion criteria involved the application of an AI model developed by the researchers to patients with GBM. The focus was on using the AI model for surgical decision-making, specifically assessing resectability and estimating surgery-related neurological outcomes and complications. Out of the 117 studies generated, only 10 studies were included in this study.

## Results

Studies utilizing AI tools in surgical decision-making are grouped under 2 headings: predicting resectability and predicting postoperative complications and neurological outcome. The studies are listed in Table 2.

**Table 2:** Studies utilizing AI tools in surgical decision-making.

Author, Year	Data	AI model description
Fathi-Kazerooni, 2015	MRI	DL for segmentation, using advanced NN to delineate tumor boundaries
Marcus, 2020	MRI	Artificial NN to recognize complex patterns in imaging data
Kommers, 2021	MRI	Deep NN for enhanced segmentation, along with sophisticated algorithms for tumor and tissue differentiation
Zanier, 2023	*Multimodal Brain Tumor Segmentation Challenge (BraTS)*	ML for tumor segmentation
Yeung, 2021	Connectome	ML with a connectome-based approach, analyzing brain network connectivity to assess tumor impact
Osipowicz, 2023	Connectome	Connectomics software [for analyzing T1 MRI, DWI and resting-state fMRI data and Hollow-tree Super (HoTS) method for mapping and analyzing abnormal brain connectivity]
Morell, 2022	Connectome	Connectomics platform, a ML tool for comprehensive brain network mapping and analysis to make surgical decisions
Luckett, 2023	Resting State fMRI	3D convolutional NN for fMRI data, extracting features from brain activity patterns to predict surgical outcomes
Caverzasi, 2016	Residual bootstrap q-ball fiber tracking	ML for DTI analysis for surgical planning
Ille, 2022	nTMS, Connectome	Integrated ML for non-invasive mapping with nTMS and connectome data to enhance surgical accuracy

ML, Machine learning; DL, Deep Learning; NN, Neural network; MRI, Magnetic resonance imaging; DWI, diffusion weighted imaging; DTI, Diffusion tensor imaging; fMRI, Functional Magnetic resonance imaging; nTMS, Navigated transcranial magnetic stimulation.

### Predicting resectability

Numerous prior studies conducting volumetric assessments and assessing resection extent have concentrated on the percentage of the removed tumor volume. A classification system that integrates both relative tumor reduction and absolute residual tumor volume has been suggested. However, there is a presumption that the absolute residual volume might carry more significance as a prognostic factor than the relative reduction of tumor volume. In 2022, the Response Assessment in Neuro-Oncology (RANO) Resect Group introduced a revised classification system, which, in contrast to the earlier systems, incorporates only absolute residual tumor volumes. Upon application of the resulting extent of resection (EOR) classification system, distinct survival outcomes were observed among the respective categories. Patients stratified into “supramaximal contrast enhancing (CE) resection” demonstrated superior outcomes compared to those with “maximal CE resection,” with the latter group being superior to patients with “submaximal CE resection.” Patients designated as “biopsy” exhibited the least favorable progression-free survival (14). The findings of this study offer a basis for developing an AI-powered prediction system to evaluate the extent of resection in gliomas.

Segmentation proves valuable not only for evaluating tumor borders but also for AI tools to efficiently segment and quantify the volume of both CE and non-CE areas of GBMs. An integral aspect of image processing in GBM, characterized by heterogeneity, is the precise segmentation of distinct tumor components, including viable tumor, edema, and necrosis. Fathi Kazerooni et al. (15) utilized a semi-automatic multi-parametric approach, integrating anatomical magnetic resonance imaging (MRI) with physiological modalities like diffusion-weighted imaging (DWI) and perfusion-weighted imaging (PWI). Thirteen GBM patients underwent T2-weighted imaging, PWI, and DWI. The spatial fuzzy C-means algorithm combined with region growing enhanced the delineation of pathogenic regions. The multi-parametric approach, coupled with semi-automatic segmentation, demonstrated a sensitivity, specificity, and dice score exceeding 80%, showcasing its potential for precise tumor characterization and efficient pre-surgical treatment planning.

Marcus et al. (16) developed a grading system based on preoperative MRI features to predict surgical resectability in gliomas. The study utilized an artificial neural network (NN) for improved prediction compared to traditional methods. The grading system incorporated anatomical features from pre-operative MRI, including the contrast-enhancing tumor was within 10 mm of the ventricles; bilateral location if the contrast-enhancing tumor extended into the corpus callosum; eloquent location if the tumor extended into motor or sensory cortex, language cortex, insula, or basal ganglia; large size if the diameter exceeded 40 mm; and associated edema if hypointensity extended more than 10 mm from the contrast-enhancing tumor. Each feature was equally weighted, and lesions were categorized based on the sum of points; as low (0–1 points), moderate (2–3 points), or high complexity (4–5 points). The study demonstrated varying rates of complete removal of CE tumors, ranging from 3.4% in high complexity lesions to 50.0% in low complexity lesions. Despite study limitations, including a small dataset and retrospective design, the authors believe the ANN can aid surgical decision-making and contribute to more meaningful comparisons in future research.

Kommers et al. (17) proposed a Standardized Glioblastoma Surgery Imaging Reporting and Data System (GSI-RADS) based on an automated method of tumor segmentation to provide standardized reports on tumor features relevant for GBM surgery. Tumor parts were segmented using both a human rater and an automated algorithm, and the extracted tumor features were compared. The study demonstrated agreement between automated and manual segmentations in various tumor features, including laterality, contralateral infiltration, tumor volumes, multifocality, location profiles, residual tumor volumes, resectability indices, and tumor probability maps via an open access software.

Zanier et al. (18) developed and validated a ML model for segmentation on MRI scans, enabling the assessment of percentage-wise tumor reduction post-intracranial surgery for gliomas. The preoperative segmentation model (U-Net) utilized MRI scans from 1,053 patients from the Multimodal Brain Tumor Segmentation Challenge (BraTS) 2021 and those who underwent surgery at the University Hospital in Zurich. Evaluation was conducted on a holdout set of 285 images from the same sources. The postoperative model was created with 72 scans and validated on 45 scans from the BraTS 2015 and Zurich dataset. The algorithm determined the extent of resection in 44.1% of the cases.

The conventional strategy in surgical neuro-oncology aims to preserve function in eloquent areas, primarily within the left dominant hemisphere to prevent aphasia. In non-eloquent regions, particularly outside the left perisylvian areas, surgery is often performed in asleep patients, potentially utilizing motor-evoked potentials to prevent hemiplegia in cases involving or near the central area. Surgical selection and planning traditionally focus on the local topography of the glioma, with limited considerations for the entire brain circuitry. However, the emerging field of mapping macro-scale neural connectivity has led to a reevaluation of classical cognitive models. This paradigm shift advocates moving from a localized understanding of brain processing to adopting a meta-networking theory of cerebral functions (19). Adopting the perspective of dynamic interactions characterized by fluctuations between segregation and integration in functional connectivity, contemporary surgical neuro-oncology is oriented toward achieving a connectome-based resection (20). This entails removing the diffuse neoplasm until real-time detection of critical cortico-subcortical circuits that underlie various functions such as movement execution, somatosensory feedback, visual function, visuospatial cognition, language (including articulatory, phonological, verbal semantic, and syntactic processing), and higher cognitive functions like executive functions (notably working memory and mental flexibility), multimodal semantics, and mentalizing. A notable insight is the substantial variability observed at the cortical level, contrasting with minimal variability at the subcortical level. A two-level model of inter-individual variability proposed by Duffau is characterized by high cortical variation and low subcortical variation suggests careful assessment of connectomics for surgical planning (21).

Many researchers performed AI tools to predict the resectability based on connectomics to better assess the postoperative neurological outcome. Connectomics, the investigation of the brain’s entire neural connections, known as the ‘connectome,’ is centered around the complex white matter pathways responsible for transmitting information between cortical and subcortical structures. The initiation of the Human Connectome Project (HCP) in 2010 has been a driving force behind the surge in interest in connectomics within both cognitive neuroscience and neurosurgery, serving as a watershed moment, instigating extensive exploration into the realm of functional brain connectivity. The persistent effort to unravel the intricate functional networks and connections within the nervous system remains an area of compelling potential for glioma surgery (22).

The definition of eloquence in neurosurgery has evolved, with the primary objective of brain tumor surgery being the optimal balance between oncologic treatment and preserving neurological function (23). While maximizing tumor resection enhances survival, the occurrence of new postoperative neurological deficits diminishes quality of life and overall survival (24–26). Traditional preoperative mapping methods focus on eloquent areas such as language, visual, and sensorimotor networks. However, these techniques, including diffusion tensor imaging (DTI), navigated transcranial magnetic stimulation (rTMS), and functional MRI, present logistical challenges and require specialized personnel (27). Beyond traditional eloquent areas, non-traditional regions affecting personality, executive function, visuospatial abilities, metacognition, semantic memory, and other cognitive functions also impact patients’ quality of life. Understanding and preserving these non-traditional eloquent areas, encompassing salience, default mode, limbic, central executive, and dorsal attention networks, is crucial. Moving beyond a localizationist paradigm, the modern brain mapping approach recognizes function within large-scale brain networks and sub-networks, rather than fixed anatomical areas. To minimize the risk of neurologic deficits, it is imperative to develop mapping tools capable of identifying both traditional and non-traditional eloquent areas. Quicktome™, a novel cloud-based platform utilizing machine-learning and reparcellation techniques, addresses the limitations of existing technologies by accurately mapping brain networks in anomalous anatomy, such as brains with tumors. Quicktome™ was developed through the integration of machine-learning techniques to produce reliable visualizations of crucial brain networks. These visualizations can be utilized in conjunction with standard neuronavigation, aiming to reduce the occurrence of deficits (28, 29).

The potential of Quicktome™ appears promising for evaluating the influence of brain tumors on large-scale networks, including both traditional and non-traditional eloquent areas, during preoperative planning. Morrell et al. (30) used this machine-learning platform to evaluate eloquent brain regions in patients undergoing brain tumor resection, employing a thorough analysis of large-scale brain networks. Of the 100 participants, the central executive network exhibited the highest incidence of alteration (49%), followed by the default mode (43%) and dorsal attention networks (32%). Notably, patients with preoperative deficits demonstrated a significantly higher number of affected networks compared to those without deficits (average 3.42 vs. 2.19, *p* < 0.001). Moreover, individuals without neurologic deficits manifested 2.19 affected and 1.51 at-risk networks, predominantly associated with non-traditional eloquent areas (*p* < 0.001). Even in patients lacking evident deficits on standard neurologic exams, non-traditional eloquent areas were frequently affected. Integrating machine-learning techniques for non-invasive brain mapping into clinical practice holds promise for preserving higher-order cognitive functions linked to these affected networks in neuro-oncology patients.

Luckett et al. (31) introduced a 3D Convolutional Neural Network (3DCNN) designed for mapping language and motor resting-state networks using minimal resting-state functional MRI (RS-fMRI) data. The 3DCNN, trained on diverse datasets, demonstrated a robust 96% out-of-sample validation accuracy. Control data comparisons revealed an impressive 97.9% similarity in mappings with 50 or 200 RS-fMRI time points. In patients with GBM multiforme, the 3DCNN accurately mapped language and motor networks, showcasing its effectiveness in presurgical planning. The study revealed the AI potential of the 3DCNN in revolutionizing preoperative planning for GBM multiforme resection, emphasizing the significant reduction in scan time and improved surgical outcomes.

Cepeda et al. (10) assessed a predictive model for identifying future recurrence areas in GBM using voxel-based radiomics analysis of MRI data. Conducted across multiple institutions, the retrospective analysis included GBM patients who underwent complete resection of enhancing tumors, with 55 meeting the study criteria. The cohort was divided into training (*N* = 40) and testing (*N* = 15) sets. Follow-up MRI provided ground truth for defining recurrence, while postoperative multiparametric MRI enabled extraction of voxel-based radiomic features. Deformable co-registration aligned MRI sequences, facilitating segmentation of the peritumoral and enhancing tumor regions. Voxels overlapping between these areas were labeled as recurrence, others as nonrecurrence. Four machine learning classifiers were trained, with the Categorical Boosting (CatBoost) model achieving the best performance on the test set (AUC = 0.81 ± 0.09, accuracy = 0.84 ± 0.06) using region-based evaluation. The study demonstrated accurate prediction of future recurrence regions, suggesting potential benefits for optimizing surgical and radiotherapy strategies to enhance patient survival in glioblastoma.

### Predicting postoperative complications and neurological outcome

Although complete resection is linked to improved survival, it poses risks of neurological deficits, occurring in approximately 1 in 10 patients (32). A crucial determinant of survival outcomes is the development of new postoperative neurological impairments, especially among patients aged over 60, those experiencing at least one new impairment exhibited the poorest survival outcome (median of 11.6 months), whereas those without new impairments achieved the best outcome (median of 28.4 months) after the complete resection of contrast enhancing tumor (24). The repercussions of these deficits can be profound, impacting both the quality of life and, ultimately, the survival of individuals affected by GBM. Choosing a universally applicable surgical modality stands as the first crucial step in the treatment of gliomas.

Predicting the outcome of a surgical procedure is a multifaceted and intricate decision-making process that takes into consideration various parameters. This encompasses factors specific to the tumor, individual patient characteristics, elements related to the health system, and considerations tied to the surgeon’s expertise. These considerations encompass the accessibility of surgical tools, the patient’s frailty, neurological condition, existing comorbidities, and even psychological aspects. Additional factors such as geographical location, ethical and social considerations, healthcare and malpractice systems, and the availability of post-operative management and care by the neuro-oncology team add layers of complexity to this prediction. Surgeon-related factors also play a crucial role in this comprehensive assessment. The surgeon’s experience and their approach to the functional neurooncology concept are pivotal elements in this convoluted assessment. Gerritsen et al. (2) conducted a survey with 224 responses from neurosurgeons across 41 countries, predominantly male (90.2%) and with diverse practice settings. The study revealed significant differences in decision-making processes among neurosurgeons, particularly between academic and non-academic/private practice respondents and European vs. US neurosurgeons. Key factors influencing treatment choice for GBM patients included tumor location, preoperative patient functioning, and neurological morbidity. While most agreed on resection followed by adjuvant therapy as the best choice, nearly a quarter favored biopsy in older patients, citing a perceived risk of morbidity outweighing survival benefits. Perioperative factors influencing an aggressive or defensive approach varied based on surgeon experience, practice setting, and geographical location. Tumor location and eloquence were deemed crucial factors, with differences observed in responses related to the location of tumors in or near eloquent areas. The study emphasized the impact of multidisciplinary neuro-oncology tumor boards and highlighted varying perspectives on age-related considerations in GBM surgery.

Efforts to formulate the decision-making process have been made in the literature. Ferroli et al. (33) devised the Milan Complexity Scale as a result of a study that evaluated consecutive elective tumor resection surgeries. This scale aims to predict neurological clinical deterioration post-surgery, incorporating factors such as tumor size, cranial nerve manipulation, brain vessel manipulation, posterior fossa location, and involvement of eloquent areas. The retrospective study, involving 746 patients with meningiomas and GBMs, produced a grading scale ranging from 0 to 8, where higher scores suggest a potential worse clinical outcome. No AI-based system has utilized this score to predict postoperative outcomes, and its applicability may be further challenged by the evolving definition of “eloquence.”

Our search revealed only two studies to predict postoperative complication or surgical outcome by AI tools.

Caverzasi et al. (34) used a residual bootstrap q-ball fiber tracking to map language pathways and rated tract injury impact on language function after glioma resection. Residual bootstrap q-ball fiber tracking was used to segment eight language pathways in 35 glioma patients. The rating scale for pathway damage significantly correlated with language performance. Preservation of the left arcuate fasciculus and superior longitudinal fasciculus correlated with no long-term deficits, while damage ensured deficits. The authors predict long-term language deficits post-surgery based on white matter tract integrity.

Ille et al. (27) enrolled 60 non-aphasic patients with left hemispheric perisylvian gliomas to investigate the prediction of surgery-related aphasia (SRA) based on function-specific connectome network properties under different fractional anisotropy thresholds combining navigated transcranial magnetic stimulation. Preoperative connectome analysis helped predict SRA development with an accuracy of 73.3% and sensitivity of 78.3%. This study provided a new perspective of function-specific connectome analysis to investigate language function in neurooncological patients. A preoperative connectome analysis seems promising to perform risk assessments predicting the development of postoperative neurological deficit.

## Discussion

The optimal surgical approaches for GBM remain a subject of ongoing debate among surgeons due to the intricate heterogeneity of gliomas, including factors such as location, grade, and patient-specific considerations. The AI tools have emerged as transformative elements in addressing these challenges, enhancing resectability and outcome prediction by capturing intricate relationships among variables. Real-time decision support, integrating automated segmentation systems with immediate feedback during preoperative and intraoperative evaluations, is a groundbreaking concept empowering neurosurgeons to make informed decisions based on imaging data.

The advancement and deployment of advanced AI algorithms are pivotal in enhancing imaging capabilities for GBM surgery. These algorithms excel in precisely delineating tumors and continuously refining their performance through learning from diverse datasets. The utilization of DL techniques becomes essential for managing the intricate patterns and variability inherent in GBM imaging. Integration of multimodal imaging, incorporating data from functional MRI, diffusion tensor imaging (DTI), and positron emission tomography (PET), offers a comprehensive perspective of tumors and surrounding structures, thereby enhancing diagnostic precision and treatment strategies. Bianconi et al. (35) demonstrated the effectiveness of an automated U-Net algorithm for GBM segmentation in clinical MRI datasets, both before and after surgery. Their validated approach addresses challenges such as low-quality imaging and improves the reliability of postoperative assessments, crucial for advancing surgical planning and prognostic predictions in neuro-oncology.

While preoperative connectome analysis holds promise for predicting the risk of neurological deficits before surgery, the challenge lies in developing an AI system to evaluate postoperative complications and neurological outcomes. Creating such a system would provide neurosurgeons with access to an evidence-based therapeutic blueprint tailored to the diverse needs of individual patients. Future AI models in intracranial tumor surgery may draw insights from existing literature based on surgeons’ predictions for surgery related neurological outcomes and postoperative complications. The dependence on surgeons’ predictions, whether through AI tools or other methods, presents a fundamental flaw. Currently, there is a lack of studies, including those involving AI tools, specifically focused on developing a tool for predicting postoperative deterioration or surgical outcomes. Surgeons typically make treatment decisions based on factors such as tumor location, size, and interaction with surrounding structures. In a prospective study involving 299 patients undergoing intracranial tumor surgery, neurosurgeons displayed a consistent tendency to overestimate postoperative functional levels, especially regarding the ability to perform normal activities at 30 days. The assessment, using the Karnofsky Performance Scale, revealed that neurosurgeons underestimated in 15% of cases, accurately estimated in 23%, and overestimated in 62% (36). Future AI models in intracranial tumor surgery may draw from existing literature based on surgeons’ predictions for surgical outcomes and postoperative complications. However, it is noted that despite the significance of functional status, surgeons tend to exhibit an overly optimistic bias when predicting postoperative functional levels. The challenge lies in developing an AI system based on predictions with limited accuracy and value. Since surgeons often exhibit an overly optimistic bias when predicting postoperative functional levels, shared decision-making, involving patients in complex treatment choices, is considered a viable approach. Nevertheless, accurately predicting the impact and trajectory of deficits, along with their implications for the quality of life, remains a challenging endeavor (37–42). Designing an AI system to assess postoperative complications and neurological outcomes, granting neurosurgeons access to an evidence-based therapeutic blueprint for a diverse range of individual patients, presents a challenging task.

Utilizing AI tools in the education and training of residents raises additional concerns. A proactive approach is crucial to mitigate biases and enhance decision-making quality, beginning with acknowledging inherent biases in thought processes. Many clinicians, particularly during their early training years, lack formal education on the cognitive aspects of medical decision-making and bias recognition. Therefore, the introduction of training programs, especially in graduate medical education, becomes essential. These programs should empower physicians to identify cognitive biases, understand decision-making processes, and reflect on past errors. Integrating AI into such programs could further enhance bias recognition and decision-making by providing data-driven insights, potentially transforming the way physicians navigate cognitive challenges throughout their careers (4).

### Limitations and challenges

The application of AI in neurosurgery introduces both promise and challenges. To effectively leverage AI in this field, several key considerations and hurdles must be addressed.

• Dataset Challenges:
o Extensive datasets are essential for AI training, but often lack verification in clinical settings.
o Variability in model performances and controversial findings add complexity.
• Radiomic Workflow Optimization:
o Optimizing parameters in radiomic workflows, covering tumor segmentation, feature extraction, and model training, is crucial.
o Comparing multiple ML algorithms within the same population is vital for understanding performance impacts.
• Focus on Resectability Prediction:
o Current studies predominantly focus on developing AI tools for predicting resectability.
o Surgeons base treatment decisions on factors such as tumor location, size, and interaction with surrounding structures.
• Challenges in Predicting Neurological Outcomes:
o AI faces hurdles in predicting postoperative neurological outcomes.
o Data quality and quantity are critical, emphasizing the need for interpretability in medical applications.
• Surgeon Bias and Shared Decision-Making:
o Surgeons tend to exhibit an optimistic bias in predicting postoperative functional levels.
o Shared decision-making, involving patients in complex treatment choices, is considered viable.
• Clinical Validation for Generalizability:
o Clinical validation is a rigorous requirement to ensure the reliability and generalizability of AI models.
o Testing models on independent datasets and diverse patient populations is necessary.
• Collaboration and Continuous Feedback:
o Collaboration with research institutions and participation in clinical trials are imperative.
o Establishing a continuous feedback loop in AI systems is pivotal for ongoing improvements and knowledge incorporation, in accuracy, and reliability.
• Ethical and Regulatory Considerations:
o Patient privacy, transparent decision-making, and adherence to regulatory standards are critical ethical and regulatory considerations.
o Responsible integration of AI in neurosurgery is essential for patient safety and trust.
• AI in Education and Training:
o Using AI tools in the education and training of residents raises concerns without establishing formal education based on established curriculum.
o A proactive approach is crucial, starting with acknowledging inherent biases in thought processes.
o Introducing training programs, particularly in graduate medical education, becomes essential, with the potential integration of AI to enhance bias recognition and decision-making.

Addressing these challenges and considerations is essential for the successful integration of AI in neurosurgery. This involves not only technical advancements but also ethical, regulatory, and educational initiatives to ensure the responsible and effective use of AI in improving patient outcomes.

### Future directions

AI relies significantly on high-quality and annotated data for accurate and trustworthy predictions. Particularly, the fields of radiomics and connectomics are advancing, incorporating enhanced imaging technologies. Collaboration with research institutions and participation in clinical trial initiatives remains imperative for integrating automated segmentation systems into ongoing studies. This collaborative effort contributes valuable data to research endeavors focused on understanding GBM heterogeneity, treatment responses, and patient outcomes. Establishing a continuous feedback loop in AI systems, wherein the system learns from new patient data and outcomes, is pivotal. This iterative process leads to ongoing improvements in accuracy, reliability, and the incorporation of emerging knowledge in GBM research. Looking ahead, the goal is to enhance automated GBM segmentation and reporting systems, ultimately improving patient care and contributing to a deeper understanding of GBM radiomics and connectomics. While navigating through these challenges and considerations, the integration of AI tools in GBM management has immense potential for advancing patient care, refining treatment strategies, and contributing to the broader comprehension of surgical decision making.

## Conclusion

The primary aim is to contribute to comprehensive effectiveness research and offer valuable insights for well-informed decision-making in surgeries for GBM. An innovative AI system should seamlessly integrate imaging, radiomics, RANO criteria, resectability studies, and connectomics, along with surgery related neurological outcomes, to enhance assessment and contribute to education and training. Despite challenges, these approaches are transforming medicine, and healthcare providers should prepare for the era of AI. However, it is crucial to acknowledge that despite these advancements, the technology remains distant from replicating the nuanced and educated decision-making of an experienced neurosurgeon.

## Data availability statement

The raw data supporting the conclusions of this article will be made available by the authors, without undue reservation.

## Author contributions

MM: Conceptualization, Data curation, Formal analysis, Investigation, Methodology, Supervision, Visualization, Writing – original draft, Writing – review & editing. MZ: Data curation, Investigation, Supervision, Writing – review & editing. IG: Investigation, Writing – review & editing. PF: Supervision, Writing – review & editing. FF: Investigation, Writing – review & editing. DN: Investigation, Writing – review & editing.
